# German yield and area data for 11 crops from 1979 to 2021 at a harmonized spatial resolution of 397 districts

**DOI:** 10.1038/s41597-024-02951-8

**Published:** 2024-01-19

**Authors:** Christoph Duden, Christina Nacke, Frank Offermann

**Affiliations:** Johann Heinrich von Thünen Institute, Institute of Farm Economics, Bundesallee 63, 38116 Braunschweig, Germany

**Keywords:** Environmental impact, Agroecology

## Abstract

Long time series with spatially highly resolved crop data are important for research projects on numerous future challenges in the environment and food sector. In this publication, we describe a dataset with crop-yield and area data for Germany from 1979 to 2021. The data are spatially resolved to 397 districts, which have an average size of 900 km^2^, and include the crops spring barley, winter barley, grain maize, silage maize, oats, potatoes, winter rape, rye, sugarbeet, triticale and winter wheat. The crop-yield data cover, on average, about 9.5 million hectares per year and 80% of Germany’s total arable land. The dataset contains 214,820 yield and area data points. These were obtained by collecting and digitizing crop data from multiple statistical sources and transforming the data to match the district boundaries in 2020. Potential applications of the data include the analysis of interactions between agricultural yields and environmental factors, such as weather; the validation of yield prediction methodologies or the analysis of yield-loss risks in agriculture.

## Background & Summary

The growing world population, increasing per capita food consumption, the environmental impacts of agricultural production and climate change pose challenges regarding food production. To study climate and environmental effects on food production, long yield time series with high spatial resolution are needed. Against this background, we present a dataset for crop yields for 11 crops from 1979 to 2021. This dataset has a 397-district resolution for Germany, one of the largest agricultural producers in Europe.

The dataset is available via the OpenAgrar data repository^1^. It includes the cultivars spring barley, winter barley, grain maize, silage maize, oats, potatoes, winter rape (i.e., winter oil-seed rape), rye, sugarbeet, triticale and winter wheat. In Germany, these crops cover, on average, about 9.5 million hectares (million ha) per year and over 80% of arable land. The data are spatially resolved to the NUTS 3 level (http://ec.europa.eu/eurostat/web/nuts/overview), which is a resolution to 397 districts with an average size of about 900 km^2^. In addition to yields, the dataset includes the district-level resolved area per crop in approximately every fourth year and the total arable land and district area in all years. In total, the dataset includes 214,820 yield and area data points (Table 1).

**Table 1:** Number of data points.

Variable	Available period	Raw data^a,b^		Final data^b^	
		Yield	Area	Yield	Area
Spring barley	1979–2021	16,306	6,219	14,083	3,983
Winter barley	1979–2021	17,267	6,689	14,977	4,120
Grain maize	1979–2021	9,285	3,576	9,095	3,200
Silage maize	1979–2021	16,625	6,306	14,467	4,090
Oats	1979–2021	15,672	6,174	13,757	3,965
Potatoes	1979–2021	12,274	6,080	13,363	3,884
Winter rape	1979–2021	15,459	5,073	13,664	3,580
Rye	1979–2021	15,809	6,046	13,761	3,921
Sugarbeet	1979–2021	13,476	5,158	11,664	3,288
Triticale	1999–2021	6,737	2,271	6,470	2,048
Winter wheat	1979–2021	17,538	6,628	15,233	4,095
Arable land	1979–2021	n.a.	10,532	n.a.	17,056
District area	1979–2021	n.a.	15,065	n.a.	17,056
**Total**		**156,448**	**85,817**	**140,534**	**74,286**
**Total yield and area data**		**242,265**		**214,820**	

Notes: a) The ‘Raw data’ columns show data prior to spatial district harmonization, outlier filtering and arable area imputations. b) Missing data points (44,995 in total) are not counted.

To create the comprehensive dataset, we queried 13 Statistical Offices of the Federal States and the Federal Statistical Office of Germany, as well as some raw data from Völker *et al*.^2^, merged their datasets and standardized them. We manually digitized printed data and spatially harmonized the districts so that the districts are identical over time, considering the numerous administrative district reforms enacted between 1979 and 2021, leading to over 400 changes in district structures. We filtered outliers and validated the final dataset using the official aggregated national yield and area statistics.

Our dataset and its validation complement existing data in terms of spatial coverage and resolution, as well as the crops and time period covered. It is comparable in structure to the French dataset of Schauberger *et al*.^3^, who published a dataset with crop yields for France at the NUTS 3 level for the years 1900 to 2018. We offer a dataset for Germany, one with a higher spatial resolution (the German average NUTS 3 district size is 900 km^2^, as compared to 5,675 km^2^ in France). Our dataset also includes data on rye, triticale and silage maize. Together, these additional three crops cover an average of 2.7 million ha per year in Germany (23% of Germany’s arable land). Moreover, we complement the dataset of Völker *et al*.^2^, which focusses only on winter wheat, winter barley, winter rape and silage maize in Germany from 1977 to 2020.

## Methods

In the following, we explain how we generated the comprehensive crop dataset and then harmonized its spatial resolution, filtered out its outliers and validated its values.

### Data Generation

We gathered the original crop data from public German statistical offices. These offices collect crop-yield and area data at regular intervals at the district level. The yield data are available annually. German statistical offices collect these data according to a specific estimation procedure on sampled farms in the respective districts. This data-collection procedure follows a national scheme (‘Ernte- und Betriebsberichtserstattung’^4^). Crop-area data is collected by statistical offices for all German farms in so-called national agricultural-structure surveys. These took place in 1979, 1983, 1987, 1991, 1995, 1999, 2003, 2007, 2010, 2016 and 2020.

Our research institute began collecting crop data from 13 regional statistical offices in Germany and storing it in a digitized, standardized format 20 years ago. Data, especially for the earliest years, were often only available in non-standardized printed form and had to be digitized manually (i.e., retyped to be stored electronically). Some of the historic data for Eastern Germany were added using the digitized raw data of Völker *et al*.^2^ (winter-barley yield and area in 1979 and 1980, silage-maize yield in 1979 to 1989, winter-rape yield in 1979 to 1989, winter-rape area in 1984 to 1989 and winter-wheat yield and area in 1979 and 1980).

For the years 1999 to 2021, regional statistical offices made parts of the data available online at Regionalstatistik^5–7^, the common data platform of German regions. Nevertheless, Regionalstatistik covers only 33% of the total yield and area data points compiled in our dataset and 72% for the years after 1999. In addition to crop data, we included data on the total district size from the federal statistical office in the data set^8,9^. Detailed references are provided in the supplementary information.

The available data on potato yields involved a challenge: the original values of the total potato yield, which is the area-weighted mean of the early and late potato yield, is incomplete before 1999. For 10% of total-potato-area observations, there are no early potato yields available in this period. Therefore, we approximate the total potato yield. If the early potato yield is missing and the early potato area is less than 20% of the total potato area, we simply assume that the early potato yields are 22% below the late potato yield. The percentage 22% corresponds to the mean difference between the early and late potato yields in Germany. We consider this approximation appropriate because early potatoes are of low relevance in German potato production (they account for 8% of total potato area) and early and late potato yields are correlated (Pearson coefficient of 0.66). Nevertheless, the reader can obtain the raw data for potatoes prior to the approximation from our additional data file, which are stored in the data repository.

### Harmonization of the Spatial Resolution

The German administration changed the number and shapes of districts over the course of the last decades, generally with the aim of reducing the number of districts but, in some cases, also as a result of decisions to reassign (parts of) municipalities to different districts. Detailed documentation of administrative reforms at the municipality level is provided by the Federal Statistical Office of Germany^10^. As a result, the number of districts decreased from 550 in 1979 to 401 in 2021. Some districts have been reshaped multiple times. Therefore, we harmonize the data to a current and uniform geographical structure. Note that our data publication only includes 397 of the 401 German districts that existed in 2021. The remaining four districts, which are Bremen, Bremerhaven, Hamburg and Berlin, are highly urbanized metropolitan regions that are not relevant to agricultural production.

We calculated the crop-specific cultivated area of a new (child) district by summing the historic (parent) districts according to Eq. (1). We take into account the share of a district that was transferred to the new district. This is important, as in some cases, the parent district was split and migrated to different children.

1 $$are{a}_{j}^{child}=\mathop{\sum }\limits_{i=1}^{n}are{a}_{i}^{parent}\ast shar{e}_{ij}^{parent}$$

where j is an index for a child district and i in an index for a parent district.

For yield variables, we calculated the weighted mean according to Eq. 2. As in Eq. 1, we consider the share of a parent district that was transferred to the child district. Additionally, by weighting yields with the arable area (*ArabLand)*, we take account of differences in district size when calculating the mean yield.

2 $$yiel{d}_{j}^{child}=\frac{{\sum }_{i=1}^{n}\,yiel{d}_{i}^{parent}\ast ArabLan{d}_{i}^{parent}\ast shar{e}_{ij}^{parent}}{{\sum }_{i=1}^{n}\,ArabLan{d}_{i}^{parent}\ast shar{e}_{ij}^{parent}}$$

Although it would be more accurate to use the cropping area for a specific crop to weight its yield, we use the total arable area of a district because crop-specific area data were not available for each district and year. Data availability for arable area is substantially better than that for crop-specific area values. Furthermore, arable land area can be considered rather constant over time, and missing values were imputed via linear inter- and extrapolation based on the available values for neighboring years.

Detailed documentation on the spatial harmonization is included in the R code and the related Excel files (see https://git-dmz.thuenen.de/duden/harmyields_public the files ‘4_merging.R’ and ‘INPUT/shared/District reform overview.xlsx’).

### Outlier Filters

We systematically checked our dataset for obvious data errors (e.g., typing errors) by identifying extreme outliers using two criteria. First, we checked whether the yields exceed the physiologically possible yield values. Similar to Schauberger *et al*.^3^, our thresholds for maximum physiologically possible yields were 10 t/ha for winter rape; 20 t/ha for spring barley, winter barley, grain maize, oats, rye, triticale and winter wheat and 200 t/ha for silage maize, potatoes and sugarbeet. Using these criteria, we found no outliers.

Second, we classify observations as outliers if both (a) the value lies above (below) the mean value in the district across all years plus (minus) four times the standard deviation and (b) the relative deviation from the district mean is greater (lower) than the mean of the respective relative deviation across all districts in a federal state in the same year by more than four times the standard deviation. For area values, we only used criterion (a). We identified seven yield observations as outliers. We deleted these values and labelled them as outliers in a separate column.

## Data Records

In this section, we first explain how the data are stored. Then, we describe the contents of the dataset and provide some exemplary illustrations of regional yield patterns.

### Data Storage

The data are stored in the OpenAgrar data repository^1^ under the address 10.3220/DATA20231117103252-0. There are four files: the raw dataset (‘Raw_data.csv’), the final dataset (‘Final_data.csv’), a text file (‘Readme.pdf’) with explanations, variable definitions and usage notes and a list of data sources (‘Data_sources.pdf’). The repository also includes two folders with additional maps visualizing the availability of data for each crop in a year-by-year manner (‘YearMapsCropArea’ and ‘YearMapsCropYield’).

The data are provided in long format. Table 2 provides an overview of the dataset columns, and Table 3 provides additional explanations of the variable names used in the column ‘var’.

**Table 2:** Overview and explanations of the column names of the data tables ‘Raw_data.csv’ and ‘Final_data.csv’.

Column name	Explanation
district_no	Official identifier of the district according to the German Federal Statistical Office. The first two digits encode the federal states of Germany, and the remaining three digits encode the district of the federal state.
district	Official district name.
nuts_id	District identifier according to the European NUTS 3 classification scheme.
year	Year of harvest.
var	Variable name (see Table 3).
measure	Measure of the variable, being either ‘yield’ or ‘area’.
value	Value for the respective district, year, variable and measure. Yield values are reported in t/ha, and area values are reported in ha.
outlier (only in Final_data.csv)	Flags missing values that were deleted during the outlier-detection procedure (1 if outlier, else 0).

**Table 3:** Overview of variables included in the column ‘var’ in the files ‘Raw_data.csv’ and ‘Final_data.csv’.

Value of ‘var’	Explanation
sb	Spring barley
wb	Winter barley
grain_maize	Grain maize
silage_maize	Silage maize
oats	Oats
potat_tot	Potatoes
wrape	Winter rape
rye	Rye
sugarbeet	Sugarbeet
triticale	Triticale
ww	Winter wheat
ArabLand	Area of arable land
district	Total district area

### Data Description and Exemplary Data Illustrations

The dataset comprises 214,820 data points for crop yield, crop area, arable land and district area for 397 districts from 1979 to 2021 in Germany (see Table 1). The number of observations is lower in the final dataset than in the raw data set because we harmonized the data to the 2020 district structure and kept only the area observations that correspond to the years with a national agricultural-structure survey.

**Fig. 1:**
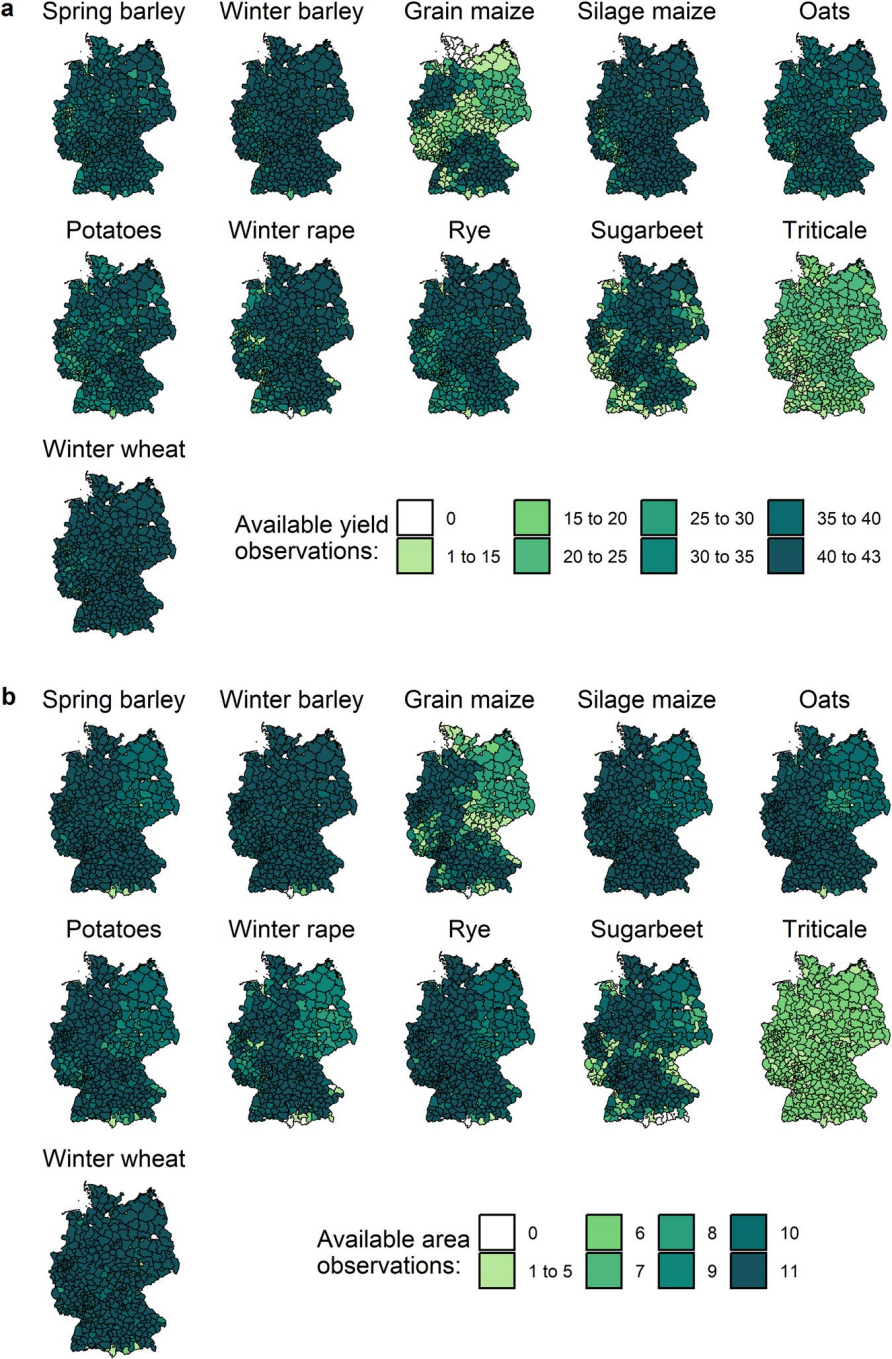
Map of German districts, with the number of available crop observations from 1979 to 2021. **(a)** Yield data. **(b)** Area data. The different colors indicate the number of available observations in the districts. Area data are only available for years with national agricultural-structure surveys (1979, 1983, 1987, 1991, 1995, 1999, 2003, 2007, 2010, 2016 and 2020).

Differentiated by districts, at least 40 years of yield observations are available for most crops (see Fig. 1a). Area data are generally available for all 11 years with a national agricultural-structure survey for most crops (see Fig. 1b). The available yield data for the selected 11 crops cover, on average, about 80% (=9.5 million ha) of total German arable land. The coverage ranges between 76% in 1996 and 86% in 2010. There are missing data points for some districts and crops (see districts with low data availability in Fig. 1; in total, there are 44,995 missing data points). As triticale was not grown on a significant scale in Germany until relatively recently, national statistics on triticale yields and areas are only available from 1999 onwards. For eastern Germany during the GDR (German-Democratic-Republic) period, regional data availability is partly limited. In particular, yield and area data for grain maize are missing for eastern Germany for the years 1979 to 1989, as grain maize was of little importance in the GDR, and area data are missing in the GDR for spring barley, silage maize, oats, potatoes, rye and sugarbeet in 1979 and for winter rape in 1979 and 1983.

**Fig. 2:**
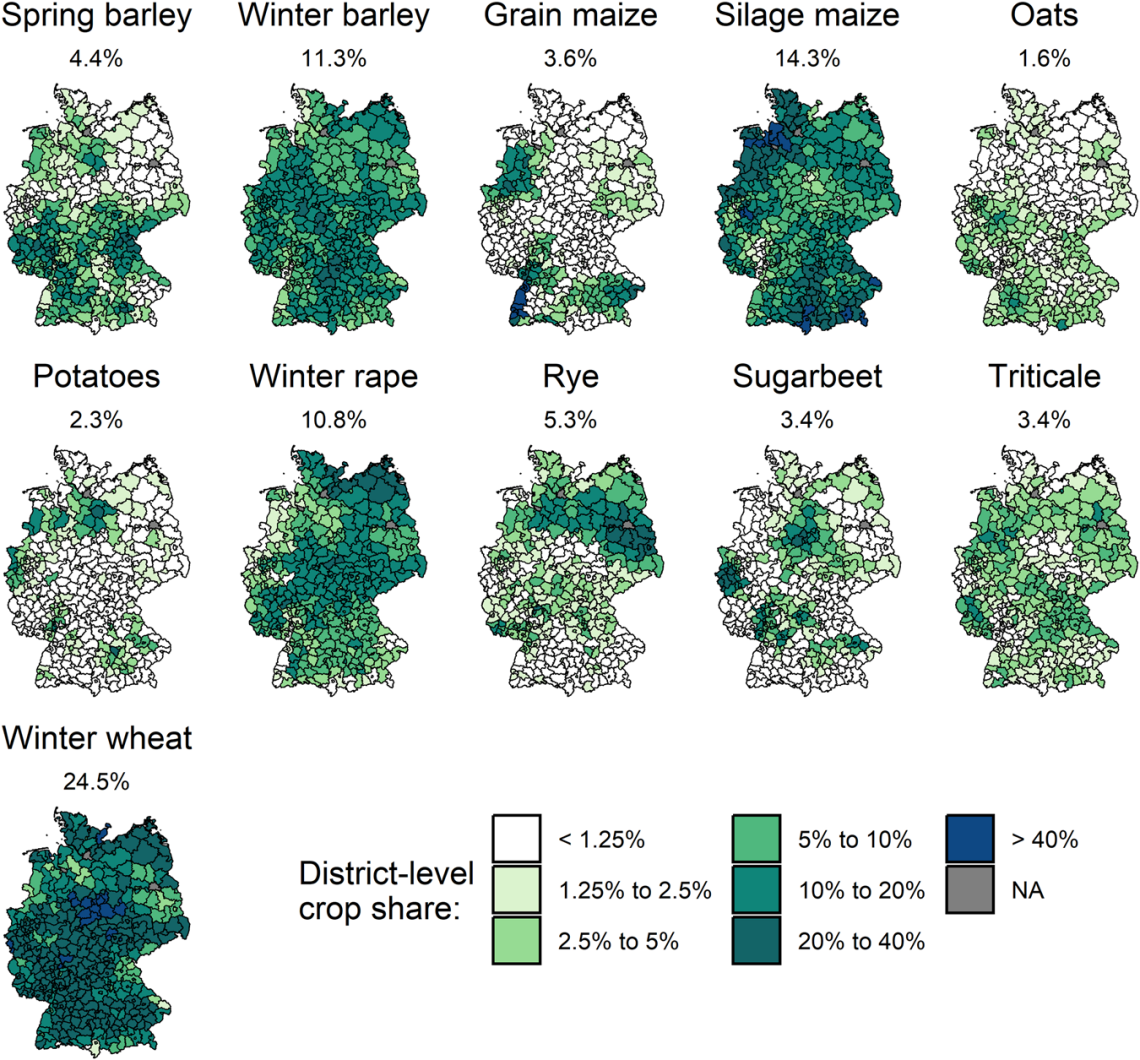
National average crop-area share with respect to arable land and its spatial variation. The different colors indicate the crop share at the district level. The crop share is calculated based on the years 1999, 2003, 2007, 2010, 2016 and 2020 (the years with agricultural-structure surveys). Moreover, for this figure, we merged our dataset with the estimated crop area data of Agraratlas (https://atlas.thuenen.de/atlanten/agraratlas) to quantify the crop share for *all* districts despite the partially low availability of official data for districts with low crop shares.

Moreover, there are also data points missing for specific crops, districts and years, mainly due to the following reasons. First, the data sources used do not consistently differentiate between a missing observation and a true zero. Second, crop yield data are not collected in a given region when the crop share is relatively low. The crop share substantially varies between crops and regions (see, e.g., grain maize and sugarbeet in Fig. 2). Third, for data-protection reasons, official authorities suppress data if the number of farms growing the respective crop in a district is low. In this case, the statistical offices often report a missing value instead of a small value or zero. Using the Agraratlas data (https://atlas.thuenen.de/atlanten/agraratlas; not public; available on request), which include estimated crop area values according to Gocht and Röder^11^, we analyzed the relevance of missing values from 1999 to 2020 in our dataset. The median area under cultivation for which yield data are missing is only 51 ha per crop and district. The median missing crop area data represent 12 ha per crop and district. These results confirm that data gaps are generally confined to districts where a given crop is of low relevance.

The standard humidity content for cereals and pulses used for grain production is 14% moisture, and for oil crops, it is 9% moisture. Yields for silage maize are reported based on 65% moisture. Potato and sugarbeet yields are reported without normalization to a standard humidity content.

The data for rye and triticale technically include both the winter and spring varieties. However, the spring varieties of these crops are of minor importance in Germany. To the best of our knowledge, there are no official statistics that report figures on each variety’s yields and cultivated area. On the basis of expert interviews and a comparison between autumn sowing estimates provided by the Federal Statistical Office^12–16^ and the harvested area for the crop aggregate, we expect the share of the spring varieties to be less than 1% of the total area under triticale or rye. Moreover, since 2010, the variable rye also includes winter maslin. Its area share is also of minor importance. Available statistics for the last year of separate data collection for winter maslin in Germany, which was 2009^17^, report a share of 1.3%.

**Fig. 3:**
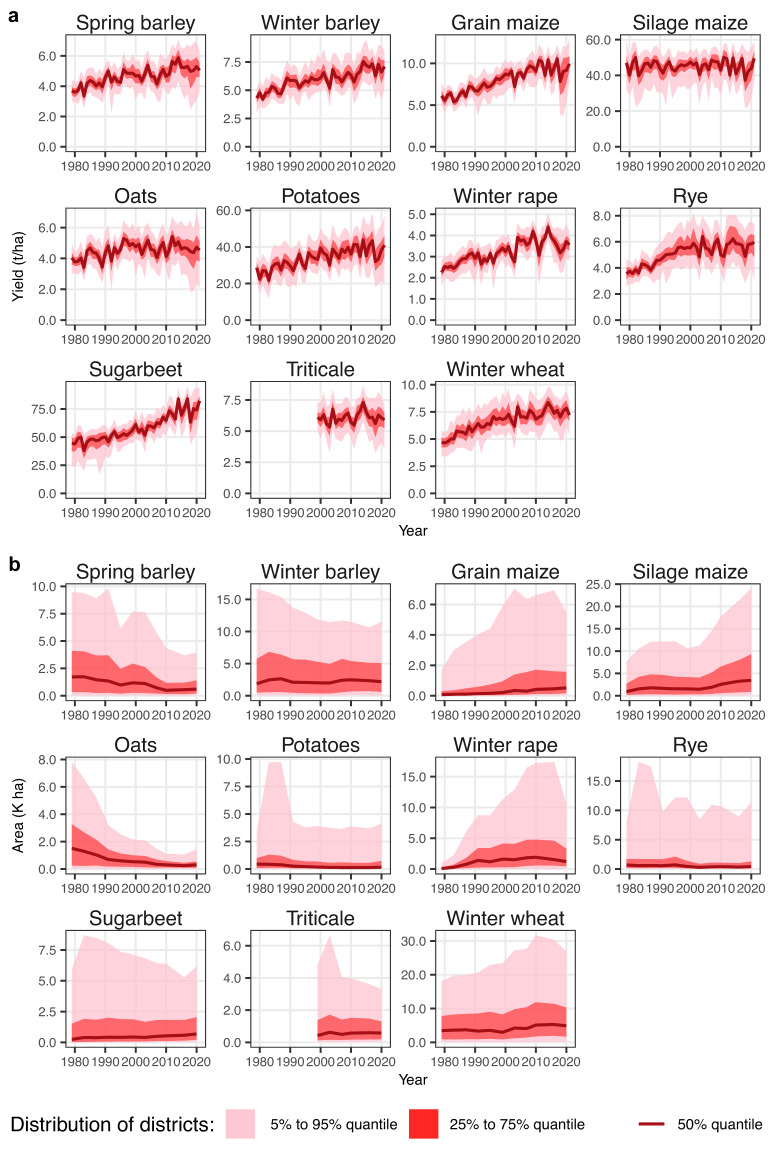
Time series of dataset values and their distribution across districts. **(a)** Yield data. **(b)** Area data. The different red shadings indicate the distribution in selected quantiles.

**Fig. 4:**
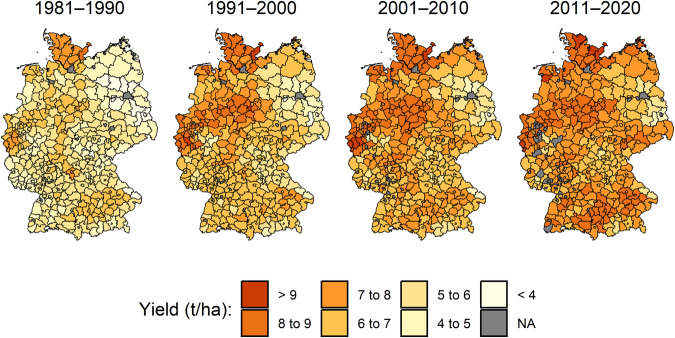
Mean winter wheat yields at the district level by decade. The different colors indicate the yield level in the districts.

**Fig. 5:**
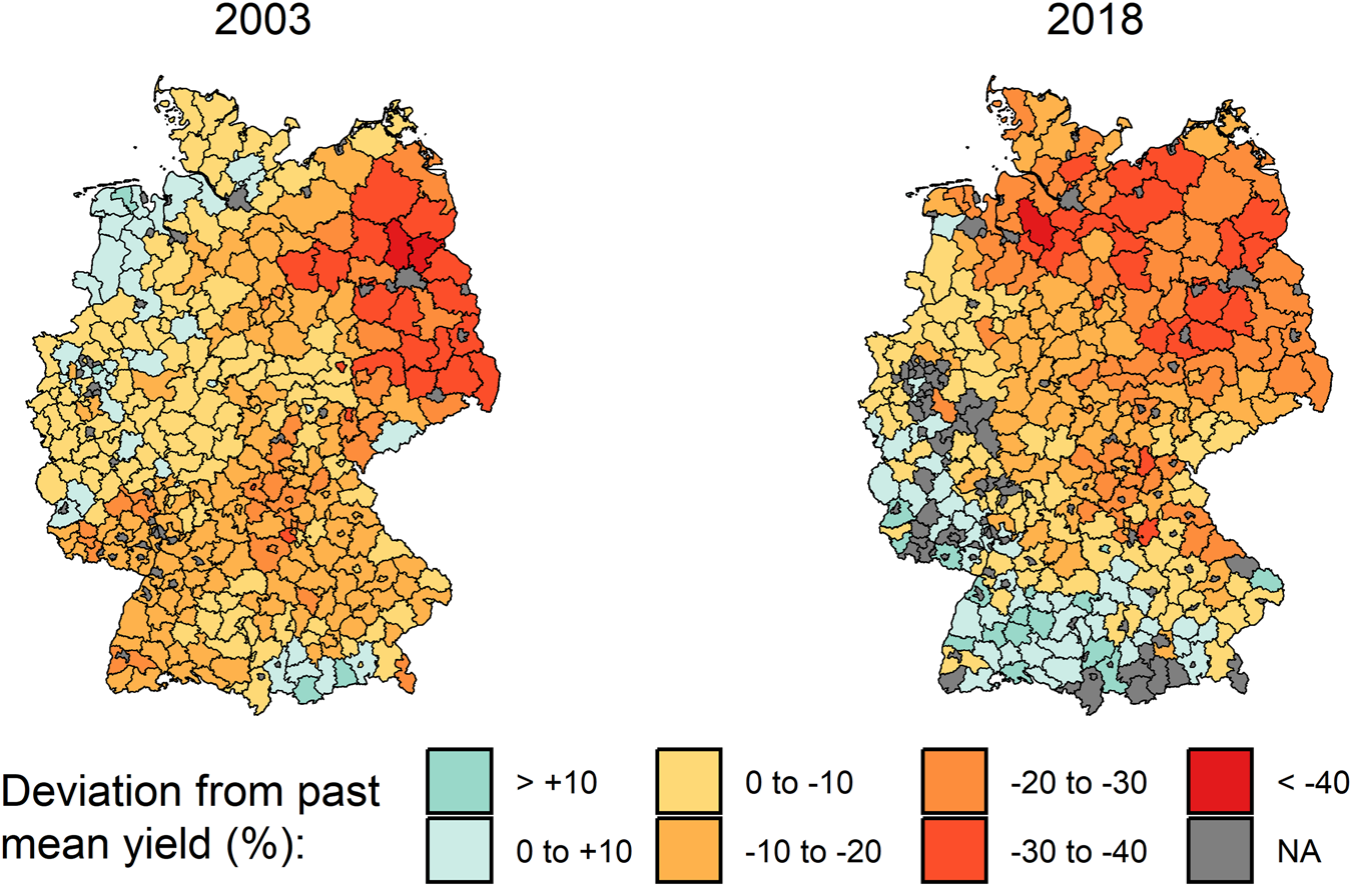
Winter wheat yields in the drought years 2003 and 2018 as deviations from the preceding 5-year average yield at the district level. The different colors indicate the level of deviation in the districts. For 2003, the preceding 5-year average is based on the years 1998 to 2002, and for 2018, it is based on the years 2013 to 2017.

In our final dataset we observe that yields show an increasing trend over time for most of the crops (Fig. 3a). Also, different yield levels are evident between districts. Moreover, the crop area varies considerably over space and time (Fig. 3b). An exemplary representation of mean wheat yields highlights the regional differences in wheat yields and the relevance of high-spatial-resolution yield data (Fig. 4). Interregional differences can be enormous in and between extreme years, demonstrating the usefulness of the yield dataset for corresponding analyses (Fig. 5). The years shown, 2003 and 2018, are considered extremely dry years in German agriculture^18^.

## Technical Validation

**Fig. 6:**
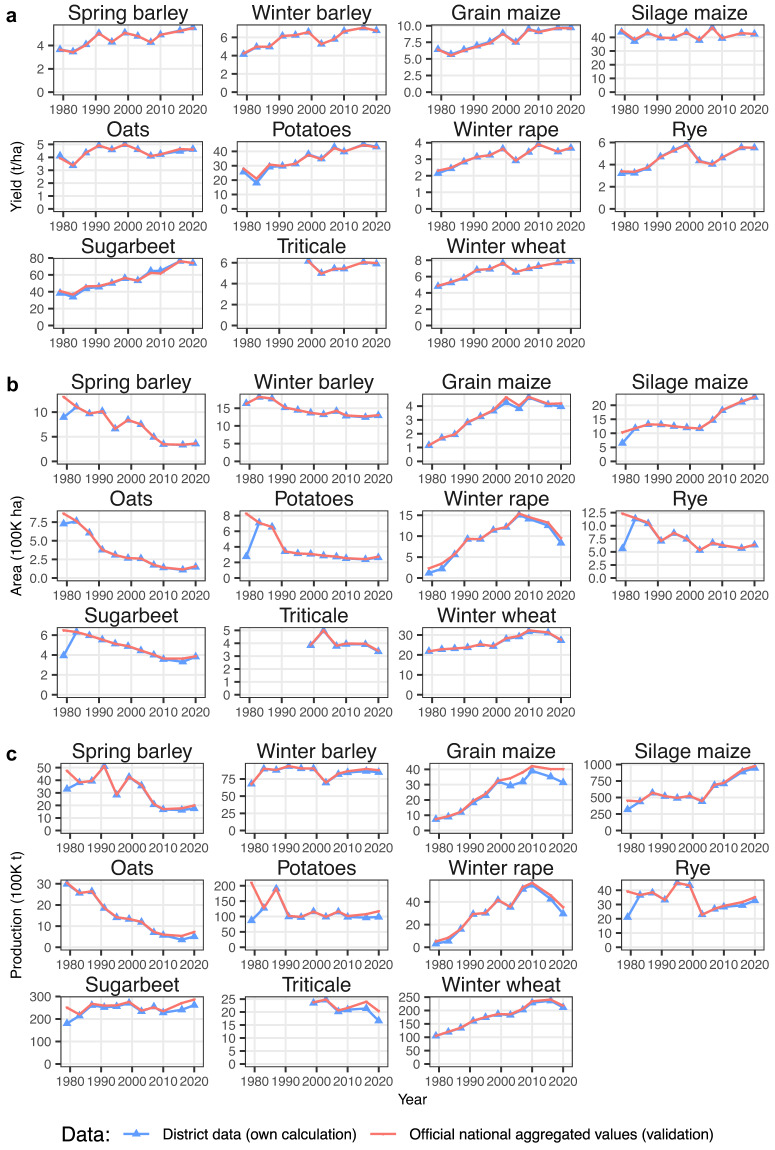
Comparison of the aggregated district data (blue line, own calculation) with the official national aggregated German crop data (red line, validation). **(a)** Yield. **(b)** Area. **(c)** Production (i.e. yield × area). For this comparison, only the years of the national agricultural-structure survey (1979, 1983, 1987, 1991, 1995, 1999, 2003, 2007, 2010, 2016, and 2020) were considered.

We validate our data by comparing them with official nationally aggregated crop-yield, area and production data (see Fig. 6). We perform this comparison for 1979, 1983, 1987, 1991, 1995, 1999, 2003, 2007, 2010, 2016 and 2020, as district-level area data are only available in these years. We calculate production as yield times area. The validation data are derived from various official sources, as single sources do not include all years, variables and crops. We used the online databases of the Federal Statistical Office and Statistical Offices of the Federal States^17,19–22^, printed publications of the Federal Statistical Office^23^ and the FAO online database^24^. As Schauberger *et al*.^3^ point out, the national validation data on yields and areas are compiled from regional information and therefore not independent from the district-level data. Nevertheless, the comparison between officially aggregated data and our district data can help reveal gaps in the dataset and errors in technical processing.

We find that the aggregated district data on yields are very similar to the validation data (Fig. 6a). Our area data are also similar to the officially aggregated area data for most years (Fig. 6b). An exception is 1979, for which are significant deviations in the areas of spring barley, silage maize, oats, rye and sugarbeet due to missing data on regional crop areas (see Data Records section). The deviations in the area data are also reflected in the production data for 1979 (Fig. 6c), as production is calculated from area and yield. Similarly, due to the lack of yield data for some districts in the period from 2010 to 2021, a slight deviation in production can be observed for oats, potatoes, winter rape, rye, grain maize, sugar beet and triticale. The reason for these recent deviations is that more and more districts with low production volumes for the respective crops are discontinuing their yield-data collection (see the Data Records section). Overall, the results indicate the high validity of the district dataset, as the few deviations from the national comparisons can be explained by missing data.

## Usage Notes

The dataset described in this data descriptor can be used by the general public if this paper and its data are cited (Creative Commons License with attribution; CC-BY 4.0).

The data are used in line with the copyright regulations of our data sources. These regulations allow for changes, editing, new designs or other amendments and distribution when the source is mentioned. The copyright regulation of the German Statistical Offices is called ‘Data license Germany – attribution – Version 2.0’, and the license text is available at www.govdata.de/dl-de/by-2-0. The data of Völker *et al*.^2^ are subject to the CC-BY regulation, and its license text is available at https://creativecommons.org/licenses/by/4.0/.

The dataset could be easily updated by integrating new data from the Federal Statistical Office and Statistical Offices of the Federal States^5,6^, as long as no new district reforms take place. However, the dataset available via the OpenAgrar^1^ data is peer reviewed in 2023 and this version will be maintained.

### Supplementary information

Supplementary Information

## Data Availability

The data were processed in R (version 4.3.2). The code to reproduce the results of this data description is publicly available at https://git-dmz.thuenen.de/duden/harmyields_public. The code is subject to the MIT license (https://opensource.org/license/mit/) and can be used freely.
