# Supplementary material for: German yield and area data for 11 crops from 1979 to 2021 at a harmonized spatial resolution of 397 districts
Source: Sci Data. 2024 Jan 19;11:95. doi: 10.1038/s41597-024-02951-8 (PMC10798971; doi:10.1038/s41597-024-02951-8)
Supplement: Supplementary file 1 — Supplementary Information [file 41597_2024_2951_MOESM1_ESM.pdf]

Supplementary Information

List of data references for
"German yield and area data for 11 crops from 1979 to 2021 at a harmonized spatial resolution of 397 districts" by
Christoph Duden, Christina Nacke and Frank Offermann,
Data Descriptor published in *Scientific Data*.

Amt für Statistik Berlin-Brandenburg. Statistische Berichte, Ernteberichterstattung über Geldfrüchte und Grünland 2007 - 2021. Table code C II 2 - j / 07 - j / 21.

Bayerisches Landesamt für Statistik und Datenverarbeitung. Statistische Beric hte, Die Bodennutzung in Bayern 1983. Table code C I 1 - j/83.

Bayerisches Landesamt für Statistik und Datenverarbeitung. Statistische Berichte, Die Ernte der Hauptfeldfrüchte und des Grünlandes in Bayern 1979 - 1998. Table code C II 1 - j/79 - j/98.

Bayerisches Landesamt für Statistik und Datenverarbeitung. Statistische Berichte, Die Ernte der Hauptfeldfrüchte und des Grünlandes in Bayern 2003, 2007, 2010, 2016. Table code C II 1 4j 2003, 4j 2007, unreg. 2010, unreg. 2016.

Bayerisches Landesamt für Statistik und Datenverarbeitung. Statistische Berichte, Die Ernte der Hauptfeldfrüchte und des Grünlandes in Bayern 2004 - 2006, 2008, 2009, 2011, 2012. Table code C II 1 j 2004 - j 2006, j 2008, j 2009, j 2011, j 2012.

Bayerisches Landesamt für Statistik und Datenverarbeitung. Statistische Berichte, Die Bodennutzung in Bayern 2003. Table code C I 1 4j 2003.

Bayerisches Landesamt für Statistik und Datenverarbeitung. Statistische Berichte, Bodennutzung der landwirtschaftlichen Betriebe in Bayern 2010, 2016. Table code C I 1 6j 2010, 6j 2016.

Bayerisches Landesamt für Statistik. Statistische Berichte, Die Ernte der Hauptfeldfrüchte und des Grünlandes in Bayern 2013 - 2015, 2017 - 2019. Table code C II 1 j 2013 - j 2015, j 2017 - j 2019.

Bayerisches Landesamt für Statistik. Statistische Berichte, Die Ernte der Hauptfeldfrüchte und des Grünlandes in Bayern 2021. Table code C II 1 j 2021.

Bayerisches Statistisches Landesamt. Allgemeine Agrarstrukturerhebung (ASE) (bis 2007), 1979, 1983, 1987, 1991, 1995. Table code 41121KX006.

Bayerisches Statistisches Landesamt. Statistische Berichte, Die Bodennutzung in Bayern 1979. Table code C I 1 - j/79.

Hessisches Statistisches Landesamt. Statistische Berichte, Agrarstrukturerhebung 2016, Landwirtschaftliche Betriebe und Bodennutzung. Table code C IV 9 - 2 - 4j/16.

Hessisches Statistisches Landesamt. Statistische Berichte, Die Bodennutzung 1980, 1983. Table code C I 1 - j/80, j/83.

Hessisches Statistisches Landesamt. Statistische Berichte, Die Getreide- und Kartoffelernte in Hessen 1982 - 1985.

Hessisches Statistisches Landesamt. Statistische Berichte, Ernteberichterstattung der landwirtschaftlichen Feldfrüchte und des Grünlands 1987 - 1998.

Hessisches Statistisches Landesamt. Statistische Berichte, Landwirtschaftszählung 2020, Landwirtschaftliche Betriebe und Bodennutzung. Table code C IV 10 - 2/20.

Information und Technik Nordrhein-Westfalen, Geschäftsbereich Statistik. Statistisches Jahrbuch Nordrhein-Westfalen 2009 - 2017. Online available at https://webshop.it.nrw.de/qsearch.php?keyword=Getreide+Anbaufl%C3%A4chen%2C+-Hektarertr%C3%A4ge+und+-Gesamtertr%C3%A4ge+2020.

Information und Technik Nordrhein-Westfalen, Geschäftsbereich Statistik. Statistische Berichte, Bodennutzung in Nordrhein-Westfalen 2010, 2016. Table code C I – j/10, j/16.

Information und Technik Nordrhein-Westfalen, Geschäftsbereich Statistik. Statistische Berichte, Ernteberichterstattung über Feldfrüchte und Grünland in Nordrhein-Westfalen 2008 - 2017. Table code C II – j/08 - j/17.

Information und Technik Nordrhein-Westfalen, Statistisches Landesamt. Statistisches Jahrbuch Nordrhein-Westfalen 2018 - 2019. Online available at https://webshop.it.nrw.de/qsearch.php?keyword=Getreide+Anbaufl%C3%A4chen%2C+-Hektarertr%C3%A4ge+und+-Gesamtertr%C3%A4ge+2020.

Information und Technik Nordrhein-Westfalen, Statistisches Landesamt. Statistische Berichte, Ernteberichterstattung über Feldfrüchte und Grünland in Nordrhein-Westfalen 2018 - 2021. Table code C II – j/18 - j/21.

Landesamt für Datenverarbeitung und Statistik Brandenburg (ab 2006 Amt für Statistik Berlin-Brandenburg. Statistische Berichte, Ernteberichterstattung über Geldfrüchte und Grünland 1991 - 2006. Table code C II 1 - j/91 - j/06.

Landesamt für Datenverarbeitung und Statistik Nordrhein-Westfalen. Statistisches Jahrbuch Nordrhein-Westfalen 2001. Online available at https://webshop.it.nrw.de/qsearch.php?keyword=Getreide+Anbaufl%C3%A4chen%2C+-Hektarertr%C3%A4ge+und+-Gesamtertr%C3%A4ge+2020.

Landesamt für Datenverarbeitung und Statistik Nordrhein-Westfalen. Bodennutzung in Nordrhein-Westfalen 2001, 2003, 2005, 2007. Table code C I – j/01, j/03, j/05, j/07.

Landesamt für Datenverarbeitung und Statistik Nordrhein-Westfalen. Statistische Berichte, Ernteberichterstattung über Feldfrüchte und Grünland in Nordrhein-Westfalen 2002 - 2007. Table code C II – j/02 - j/07.

Landesamt für Datenverarbeitung und Statistik Nordrhein-Westfalen. Statistische Berichte, Ernteberichterstattung über Feldfrüchte und Grünland in NRW, Kartoffelernte 1979, 1980, 1981, 1982, 1983, 1979 u. 1983 mit Fläche. Table code C II 1 -j/79, j/80, j/81, j/82, j/83.

Landesamt für Datenverarbeitung und Statistik Nordrhein-Westfalen. Statistische Berichte, Ernteberichterstattung über Feldfrüchte und Grünland in NRW, Endgültiges Ergebnis der Getreideernte 1979 - 1984, 1979 u. 1983 mit Fläche. Table code C II 1 - j/79 bis j/84.

Landesamt für Statistik Niedersachsen. Statistische Berichte Niedersachen, Bodennutzung und Ernte 2017 - 2021. Table code C I 1, C II 1, C II 2, C II 3 - j / 2017 - 2021.

Landesamt für Statistik Niedersachsen. Tabelle (Land und Kreise) Erträge und Fläche ab 1949 bis 2010. Online available at https://www.statistik.niedersachsen.de/landwirtschaft_forstwirtschaft_fischerei/erntestatistik_online/wachstum-und-ernte-erntestatistik-online-192533.html.

Landesbetrieb für Statistik und Kommunikationstechnologie Niedersachsen . Statistische Berichte Niedersachen Landwirtschaftszählung 2010, Heft 3. Table code C IV 9.3 - j / 10.

Ministerrat der DDR, Staatliche Zentralverwaltung für Statistik, Staatliche Zentralverwaltung für Statistik. Wichtige Kennziffern der Landwirtschaft und Nahrungsgüterwirtschaft der Bezirke der Deutschen Demokratischen Republik nach Kreisen - Hektarerträge Getreide, Winterölfrüchte, Hackfrüchte, Grün- und Silomais, Wiesen, Feldfutterpflanzen, 1979 bis 1989.

Ministerrat der DDR, Staatliche Zentralverwaltung für Statistik, Staatliche Zentralverwaltung für Statistik, Abteilung Berichtswesen der Landwirtschaft und Nahrungsgüterwirtschaft. Wichtige Kennziffern der Landwirtschaft und Nahrungsgüterwirtschaft der Bezirke der Deutschen Demokratischen Republik nach Kreisen - Landwirtschaftliche Nutzfläche, Ackerland, Grünland, Anbaufläche wichtiger Fruchtarten, 1979 bis 1989.

Niedersächsisches Landesamt für Statistik. Statistische Berichte Niedersachen, Bodennutzung und Ernte 1991 - 2021. Table code C I 1, C II 1, C II 2, C II 3 - j / 1991 - 2021.

Niedersächsisches Landesamt für Statistik. Statistische Berichte Niedersachsen Agrarstrukturerhebung 2003, Heft 3. Table code C IV 9.3 - j / 03.

Niedersächsisches Landesamt für Statistik. Statistische Berichte Niedersachsen Agrarstrukturerhebung (ASE) 2016, Heft 3. Table code C IV 9.3 - 3j / 2016.

Niedersächsisches Landesamt für Statistik. Statistische Berichte Niedersachen Landwirtschaftszählung 2020, Heft 3. Table code C IV 9.3 – 10j / 2020.

Statistische Ämter des Bundes und der Länder Deutschland, 2023. Regionalstatistik, Landwirtschaftliche Betriebe mit Ackerland und deren Ackerfläche nach Fruchtarten - Erhebungsjahr - regionale Tiefe: Kreise und krfr. Städte, Allgemeine Agrarstrukturerhebung (bis 2007). Table code 41120-02-02-4.

Statistische Ämter des Bundes und der Länder Deutschland, 2023. Regionalstatistik, Anbau auf dem Ackerland in landwirtschaftlichen Betrieben nach Fruchtarten - Jahr - regionale Tiefe: Kreise und krfr. Städte, Agrarstrukturerhebung/Landwirtschaftszählung. Table code 41141-02-02-4.

Statistische Ämter des Bundes und der Länder, Deutschland. Regionalstatistik, Erträge ausgewählter landwirtschaftlicher Feldfrüchte - Jahressumme - regionale Tiefe: Kreise und krfr. Städte, 1999 - 2021, Ernte- und Betriebsberichterstattung (EBE). Table code 41241-01-03-4.

Statistische Bibliothek. Fachserie. 3, Land- und Forstwirtschaft, Fischerei. Reihe 33, Landwirtschaftliche Bodennutzung und pflanzliche Erzeugung. 1976 bis 2014. Wiesbaden: Statistisches Bundesamt. ZDB-ID: 2175254-0. https://www.statistischebibliothek.de/mir/receive/DESerie_mods_00000037

Statistisches Amt des Saarlandes. Statistische Berichte, Agrarberichterstattung 1981. Table code C0 AB 1981.

Statistisches Amt des Saarlandes. Statistische Berichte, Bodennutzung 1979, 1983, 1987. Table code C I 1 - j. 1979, j. 1983, j 1987.

Statistisches Amt für Hamburg und Schleswig-Holstein. Statistische Berichte, Bodennutzung und Ernte 1979 - 2021. Table code CI/CII - j79 - j/21.

Statistisches Amt für Hamburg und Schleswig-Holstein. Statistische Berichte, Kreisergebnisse Schleswig-Holstein 2016. Table code C IV - ASE 2016 SH, SK Sonderbericht Kreisdaten.

Statistisches Amt für Hamburg und Schleswig-Holstein. Statistische Berichte, Kreisergebnisse Schleswig-Holstein 2020. Table code C IV - LZ 2020 SH, Sonderbericht Kreisdaten.

Statistisches Amt Mecklenburg-Vorpommern. Statistischer Bericht, Bodennutzung und Ernte 1991-2021. Table code C I - j, C II - j.

Statistisches Landesamt Baden-Württemberg. Statistische Berichte, Bodennutzung 1977. Table code C I 1 - j/77.

Statistisches Landesamt Baden-Württemberg. Statistische Berichte, Landwirtschaftszählung/allgemeine Bodennutzungshaupterhebung 1999. Table code C I 1 - 2j/99.

Statistisches Landesamt Baden-Württemberg. Statistische Berichte, Agrarstrukturerhebung/allgemeine Bodennutzungshaupterhebung 2003. Table code C I 1 - 2j/03.

Statistisches Landesamt Baden-Württemberg. Statistische Berichte, allgemeine Agrarstrukturerhebung 2007. Table code C I 1 - 2j/07.

Statistisches Landesamt Baden-Württemberg. Statistische Berichte, Landwirtschaftszählung 2010, 2020. Table code C I 1 - 2j/10, j/20.

Statistisches Landesamt Baden-Württemberg. Statistische Berichte, Agrarstrukturerhebung 2016. Table code C I 1 - j/16.

Statistisches Landesamt Baden-Württemberg. Statistische Berichte, Ernte der Hauptfeldfrüchte 1980 - 2000. Table code C II 1 - j/80 - j/00.

Statistisches Landesamt Baden-Württemberg. Statistische Berichte, Ernte der Hauptfeldfrüchte in Baden-Würtemberg 2001 - 2021. Table code C II 1 - j/01 - j/21.

Statistisches Landesamt des Freistaates Sachsen. Ernte- und Betriebsberichterstattung Hektarerträge ab 1991 -2021. Table code 41241-020Z.

Statistisches Landesamt des Freistaates Sachsen. Statistische Berichte, Bodennutzung und Ernte, 2002 -2021. Table code C II 2 - j/02 - j/21.

Statistisches Landesamt Rheinland-Pfalz. Ernteberichterstattung über Feldfrüchte und Grünland 1993 - 1998

Statistisches Landesamt Rheinland-Pfalz. Statistische Berichte, Agrarstrukturerhebung 2003, 2007

Statistisches Landesamt Rheinland-Pfalz. Statistische Berichte, Agrarstrukturerhebung - Landwirtschaftszählung 2010

Statistisches Landesamt Rheinland-Pfalz. Statistische Berichte, Agrarstrukturerhebung 2016

Statistisches Landesamt Rheinland-Pfalz. Statistische Berichte, Ernteberichterstattung über Feldfrüchte und Grünland 2007, 2010, ab 2016 jährlich

Statistisches Landesamt Rheinland-Pfalz. Statistischer Bericht, Die Landwirtschaft 1979 bis 1992

Statistisches Landesamt Saarland. Statistische Berichte, Bodennutzung 1991, 1995, 1999, 2003. Table code C I 1 - j 1991, j 1995, j 1999, j 2003.

Statistisches Landesamt Saarland. Statistische Berichte, Ernte 1979 - 1998

Statistisches Landesamt Saarland. Statistische Berichte, Ernteberichterstattung 2001 (inkl. 2000), 2002 - 2005. Table code C II 1- 4 - j 2001, C II 1 - j 2002 - j 2005.

Statistisches Landesamt Saarland. Statistische Berichte, Ernteberichterstattung 2015 (inkl. 2014). Table code C II 1 - j 2015.

Statistisches Landesamt Sachsen-Anhalt, Halle (Saale). Statistische Berichte, Ernteermittlung für Feldfrüchte und Grünland, Obst und Gemüse, 2006 - 2012. Table code C II j/06 - j/12.

Statistisches Landesamt Sachsen-Anhalt, Halle (Saale). Statistische Berichte, Wachstumsstand und Ernte, 1991 - 2021. Table code C I, C II j.

Thüringer Landesamt für Statistik. Statistischer Bericht, Ernte- und Betriebsberichterstattung Feldfrüchte und Grünland, 1991 - 2021. Table code C II - j.
